# Impact of degradation and time of sampling on gut Microbiome composition in wild-caught marine fish

**DOI:** 10.1038/s41598-025-14683-9

**Published:** 2025-08-07

**Authors:** Yufei Zhou, Alejandro Trujillo-González, Simon Nicol, Marion Boutigny, Roger Huerlimann, Stephen D. Sarre, Dianne Gleeson

**Affiliations:** 1 Centre for Conservation Ecology and Genomics, EcoDNA group, University of Canberra, 11 Kirinari Street, Canberra, ACT 2617 Australia; 2 Oceanic Fisheries Programme, Pacific Community, Noumea, New Caledonia; 3 Marine Climate Change Unit, Okinawa Institute of Science and Technology Graduate University, Onna-son, Okinawa, Japan

**Keywords:** Gut microbiome, Wild-caught fish, Katsuwonus pelamis, Microbiome degradation, Sample preservation, Microbiology, Microbial ecology, Marine biology

## Abstract

The gut microbiome has the potential to be an effective indicator of individual and population health in fish given its sensitivity to internal and external stressors. However, without consistent and tested validated sampling protocols, the gut microbiome’s potential as a reliable indicator may be limited. Routine sampling of wild free-living fish caught by commercial fisheries rarely occurs at the time of capture and more commonly occurs hours, days, or weeks after fish capture when the catch is unloaded in port. This delay in sampling provides time for the degradation and decomposition of the microbiome community potentially compromising the reliability of gut microbiome analyses. Unfortunately, comprehensive and systematic analyses on post-capture changes in the gut microbiome communities of wild marine fish are lacking, limiting the reliability of microbiome studies. Here, we investigated the post-mortem changes in the gut microbiome of one wild-caught marine fish, skipjack tuna (*Katsuwonus pelamis*), at five different time points (immediately, 2 h, 24 h, 12 days, and 24 days after fish capture). The decomposition of the gut microbiome community occurred within the first 24 h if the samples were not preserved immediately. Over time, the relative abundance of Vibrionaceae decreased while Bradyrhizobiaceae increased, indicating the potential of these two families to serve as gut microbiome degradation indicators. Our findings highlight the importance of timely preservation in gut microbiome studies of wild-caught fish. Without appropriate preservation, both the diversity of the gut microbiome and the relative abundance of key microbial families change significantly within 24 h. To obtain reliable and representative results, we recommend preserving gut samples as soon as possible after capture, ideally within two hours, and no later than 24 h.s.

**Supplementary Information:**

The online version contains supplementary material available at 10.1038/s41598-025-14683-9.

## Introduction

The gut microbiome of an individual is closely related to their health, as well as the regulation of their physiological functions^1–3^. The gut microbiome has been observed to exhibit both mutualistic and pathogenic impacts on the host, thereby playing an important role in metabolic processes, nutritional provisioning, and the functionality of immune system^2–6^. In the case of fish, previous studies have indicated significant correlations between the gut microbiome of fish and their physical condition, diet, water quality, pollution, and stress induced by human activities^3,7^. Environmental factors such as water temperature, salinity, pH, and seasonal variation have also been shown to influence gut microbial communities and diversity^8–10^. The close association between fish hosts and their gut microbiome suggests that the gut microbiome composition may provide a reliable indicator for assessing the fitness condition, environmental stress, and anthropogenic influences on fish hosts^11–13^.

One of the challenges in the microbiome assessment from gut tissue is the rapid change of the microbial community, particularly in wild-caught and warm-blooded fish species such as tunas and mackerels, following the host’s death^14^. This issue has the potential to significantly impact the accuracy of gut microbiome analyses, as microbiome composition is expected to be influenced by the process of degradation and decomposition^15–17^. However, the timing and extent of these changes has predominantly targeted mammals under laboratory conditions^15,17,18^ while remaining poorly understood in wild marine fish because of the necessity of conducting fish microbiome sampling in a manner that does not disrupt fishing operations. In particular, studies aiming to analyse gut microbiome composition of wild-caught large commercial fishes have commonly sampled fish that have been either stored on ice (often for several days)^14,19^ or are brine or ultra-frozen (often for periods of weeks to months) before they are processed^20–22^. Significant changes in the gut microbiome arising from particular preservation approaches could critically affect the ecological implications of the results arising from microbiome analyses^23–25^ so an accurate understanding of their impacts is essential.

Increasing use of gut microbiome data in ecological and environmental assessments of wild marine fish linking fish gut microbiota to host health, environmental stressors, and climate variability^12,19–22,26^ makes the limited understanding of how sampling and preservation time affect gut microbiome composition particularly concerning. Few of these studies have accounted for the potential impacts of sampling delays and preservation methods, leaving open the possibility of ecological misunderstandings and management missteps. For example, sample degradation may have reduced the ability to detect meaningful patterns between tuna gut microbiomes and El Niño–Southern Oscillation (ENSO) events in a recent study^22^, resulting in the observed lack of correlation between the two.

To analyse the gut microbiome accurately and develop reliable sampling protocols for wild-caught marine fish, it is essential to understand how the microbiome degrades and how its composition changes over time. Here, we provide empirical data on gut microbiome degradation using wild-caught skipjack tuna (*Katsuwonus pelamis*) (Linnaeus, 1758), as a representative of large marine commercial fish^27–30^. To determine the influence of decomposition in the gut microbiome community of skipjack tuna, we apply DNA metabarcoding to measure the gut microbiome decomposition in captured tuna during research sampling events. We examine gut microbiome changes at five post-capture time points (immediate, 2 h, 24 h, 12 days, and 24 days) under two experimental setups: intra-individual (sampling of the same fish at each time point) and inter-individual (sampling of different fish at each time point). This dual approach enabled us to account for both individual variation and general patterns in microbial shifts, with implications for sampling protocols in fisheries and ecological studies.

## Results

### Experiment 1 intra-individual gut Microbiome degradation comparison

A total of 49 gut samples from ten individual fish at five time intervals passed quality control and were subjected to sequencing (Additional file 1). One sample (of the original 50) was excluded due to insufficient amplification during PCR. Real-time qPCR had an average efficiency at 97.7% ± 2.5% (R^2^ = 0.936 ± 0.02, Error = 0.425 ± 0.19). No positive amplification was observed in all negative controls used across all stages of testing (i.e., sample collection, DNA extraction, and library preparation) and were not therefore included in the Illumina MiSeq sequencing. A total of 5,987,759 raw reads were obtained across all samples, with 122,199 ± 38,000 reads per sample, of which 14,978 ± 6,056 reads per sample remained after filtering, denoising, and chimera removal. A total of 4,362 ASVs were generated, wherein 4,038 ASVs could be unambiguously assigned to phylum level and passed chloroplast and mitochondrial sequence filtering. Two samples were identified as anomalies and discarded before the following analysis. Two samples were excluded from further analysis as they were dominated by a small number of bacterial families and exhibited microbial compositions highly dissimilar from all other samples in the same fish. All sequence data were uploaded to the National Center for Biotechnology Information (NCBI) and are accessible with project number PRJNA1095190 (https://www.ncbi.nlm.nih.gov/sra/PRJNA1095190).

### Time-related variance in experiment 1

**Fig. 1:**
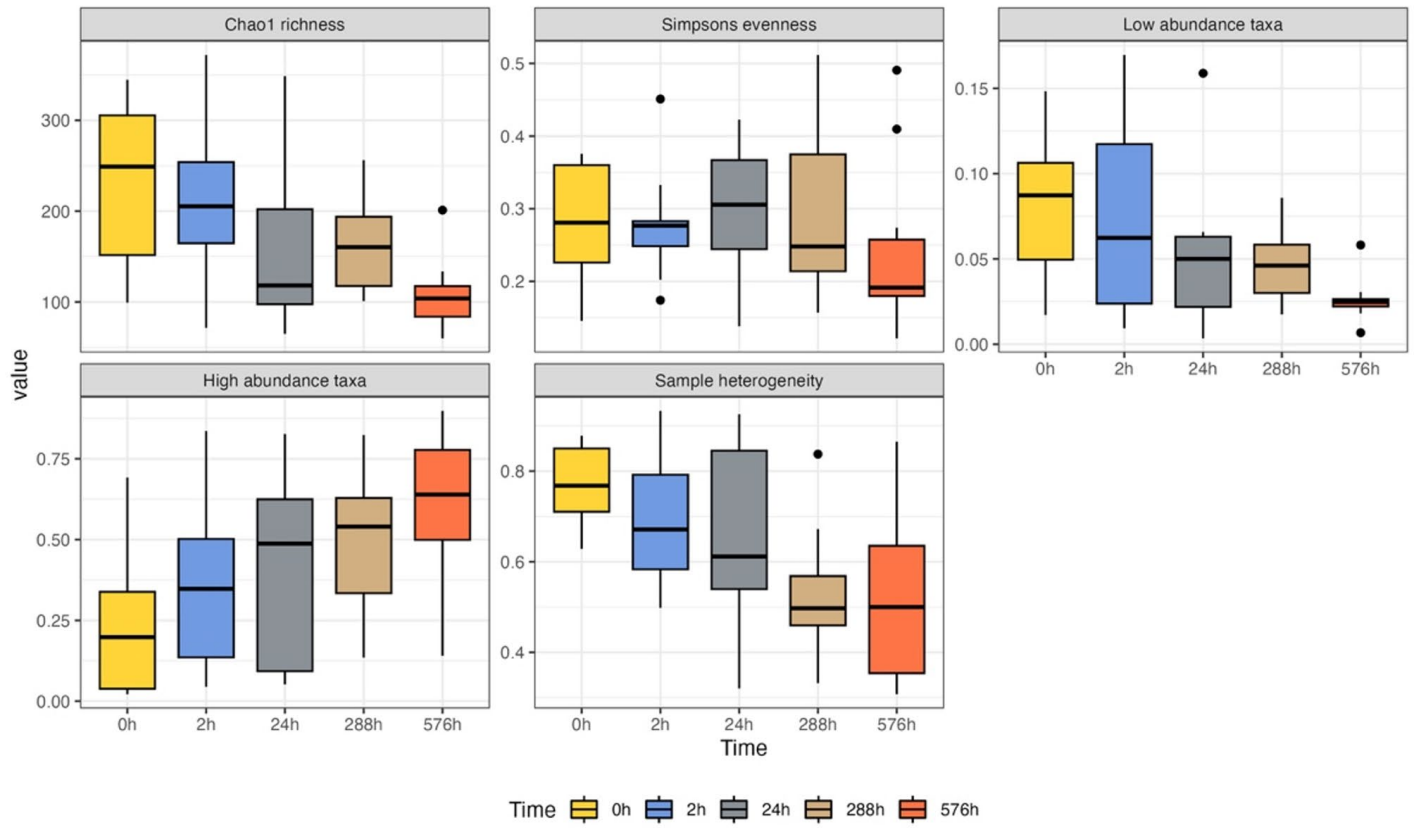
Comparison of diversity indices and sample heterogeneity across time of sampling. Y-axis for Chao1 richness and Simpsons evenness shows the respective values; Y-axis for low abundant taxa and high abundant taxa shows the relative proportion of low and high abundant taxa; Y-axis for sample heterogeneity shows the level of differences among fish individuals.

Time of sampling significantly affected Chao1 species richness (*p* < 0.01, df = 4, F = 7.9), relative abundance of rare species (*p* < 0.01, df = 4, F = 5.0), high abundant species (*p* < 0.01, df = 4, F = 9.5), and sample heterogeneity (*p* = 0.02, df = 4, F = 1.33) (Fig. 1). On the other hand, there were no significant differences in species evenness associated to time of sampling (Simpsons evenness, *p* = 0.45, df = 4, F = 0.94). Species richness and relative proportion of highly abundance taxa significantly increased/decreased over time, with a clear difference between time 0 h and 24 h (Table 1). Contrary to this, the relative abundance of lowly abundant taxa and sample heterogeneity only showed significant differences between 0 h and 576 h (Table 1).

**Table 1:** Experiment 1 diversity indices pairwise test p-values.

		Chao1 richness^1^	Low abundant taxa^1^	High abundant taxa^1^	Sample heterogeneity
0 h	2 h	0.89	0.94	0.23	0.10
	24 h	0.04*	0.24	0.03*	0.18
	288 h (12d)	0.05*	0.12	< 0.01*	0.34
	576 h (24d)	< 0.01*	< 0.01*	< 0.01*	0.01*

^1^. Tested by TukeyHSD multiple comparisons of means; Only diversity indices significantly affected by sampling time are shown.

**Fig. 2:**
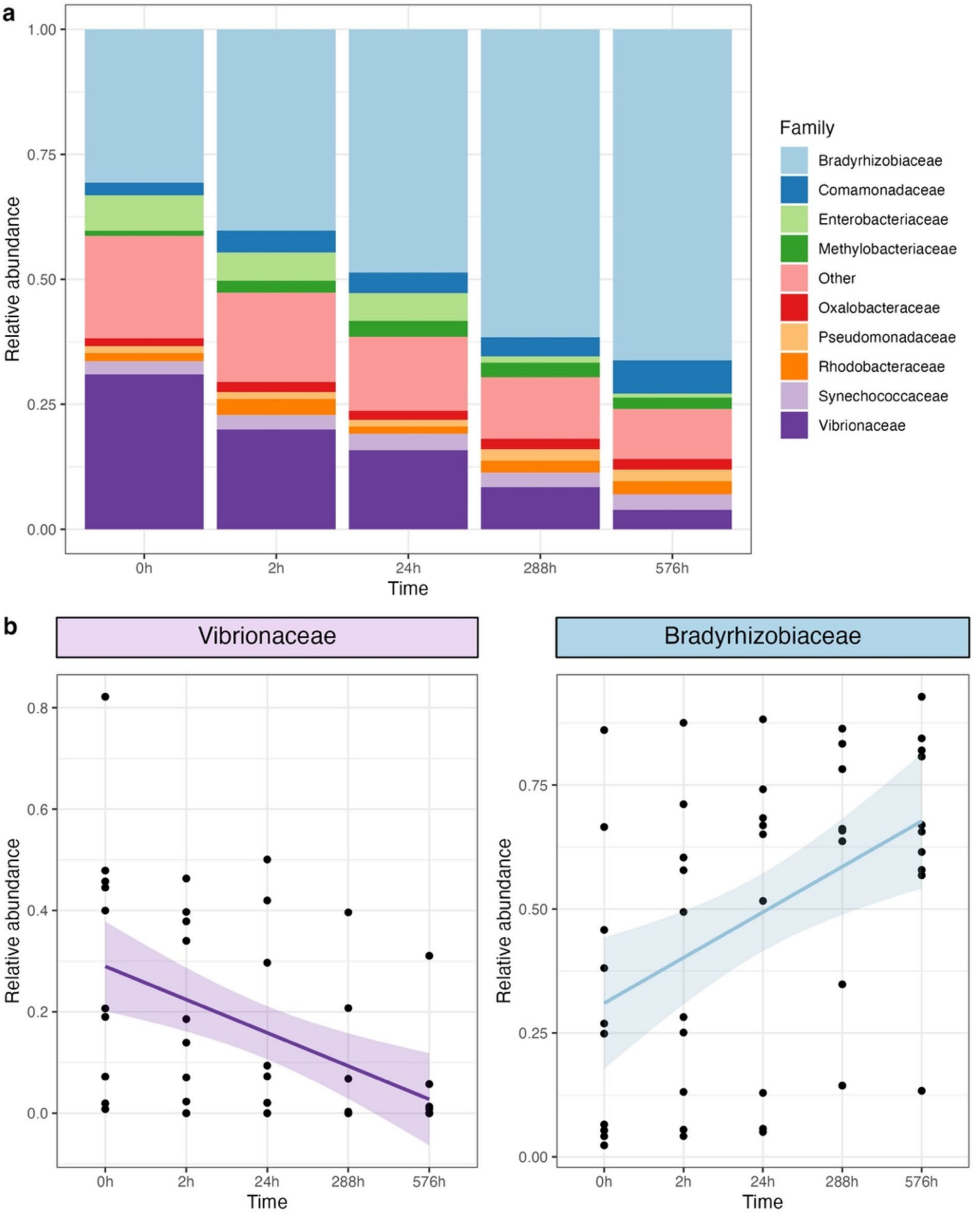
Relative abundance of the top ten most abundant families across time periods in experiment 1 (Other: rare families with detection level < 0.01) (**a**); Relative abundance change of two important families Vibrionaceae (purple, *p* < 0.01) and Bradyrhizobiaceae (blue, *p* < 0.01) (**b**).

The most abundant families in experiment 1 were Bradyrhizobiaceae and Vibrionaceae (Fig. 2a). These two families were also families with the most significant contribution to the dissimilarity between 0 h and 576 h (SIMPER: Bradyrhizobiaceae: 22%, *p* = 0.02; Vibrionaceae: 15% contribution, *p* = 0.03). The changes of Bradyrhizobiaceae and Vibrionaceae were the most significant over time, showing a decrease in Vibrionaceae (GLM: *p* < 0.01, coefficient = −0.0004, AIC = −21.1) and an increase in Bradyrhizobiaceae (GLM: *p* < 0.01, coefficient = 0.0005, AIC = 15.8) (Fig. 2b).

### Individual related variance in experiment 1

**Fig. 3:**
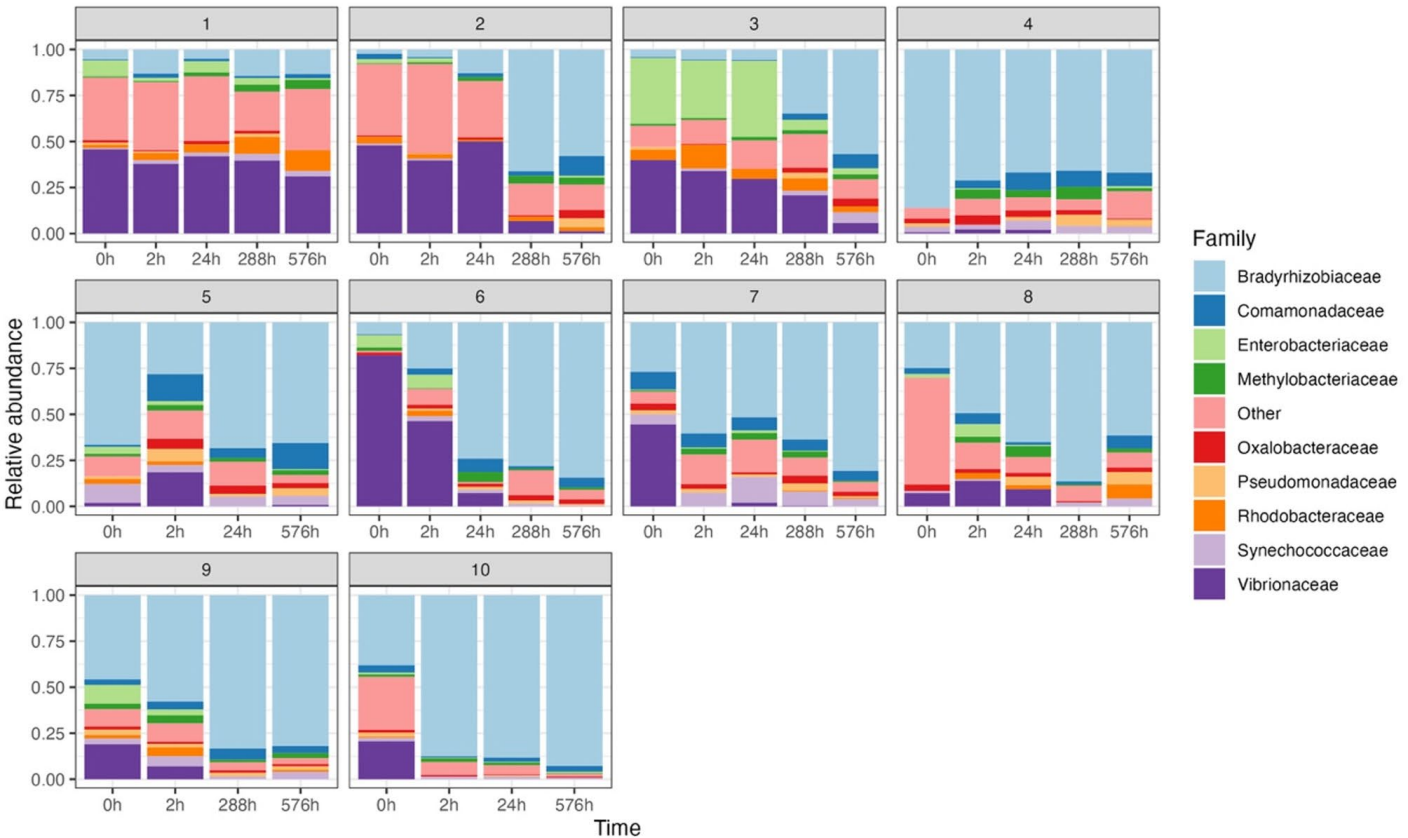
Relative abundance of the top ten most abundant families across time periods, faceted by individual skipjack tuna (Other: rare families with detection level < 0.01).

Significant individual-level differences existed in all diversity indices (Additional file 2, Table 2). The changes of Bradyrhizobiaceae and Vibrionaceae were significant while controlling individual variances (Table 2). The relative abundance of top ten most abundant families was faceted by individuals, showing the consistent changes of Bradyrhizobiaceae and Vibrionaceae across all individual except for Fish 1 and Fish 4 (Fig. 3). Fish 1 showed a stable microbiome composition across time periods, with no significant decrease of relative abundance in Vibrionaceae (GLM: *p* = 0.17, AIC = −10.6) nor increase in Bradyrhizobiaceae (GLM: *p* = 0.30, AIC = −19.6). While fish 4 showed a high relative abundance of Bradyrhizobiaceae in all time periods from the beginning (0 h), and the relative abundance of Bradyrhizobiaceae remained stable along with time (GLM: *p* = 0.63, AIC = −6.80).

**Table 2:** ANCOVA test result with time as explanatory variable, relative abundance of vibrionaceae and Bradyrhizobiaceae as response variable, and fish individual variance as covariant.

	Exploratory factors	Df	F-value	Pr (> F)
Vibrionaceae	Time	4	19.3	< 0.01*
	Fish	9	4.86	< 0.01*
Bradyrhizobiaceae	Time	4	17.6	< 0.01*
	Fish	9	7.07	< 0.01*

### Experiment 2 inter-individual gut Microbiome degradation comparison

Of 50 total fish gut lining samples, six samples were lost during sample processing. The remaining 44 passed quality controls and were sequenced (Additional file 1). Real-time PCR had an average efficiency of 90.3% ± 2.9% (R^2^ = 0.957 ± 0.04, Error = 0.276 ± 0.13). No positive amplification was observed in all negative controls used across all stages of testing (i.e., sample collection, DNA extraction, and library preparation) showed and so no controls were included in the Illumina MiSeq sequencing. A total of 5,696,099 raw reads were generated, with 129,457 ± 45,137 reads per sample. After filtering, denoising, and chimera removing, 16,940 ± 6,955 reads per sample left. A total of 4,874 ASVs were generated, wherein 4,574 ASVs could be unambiguously assigned to phylum level and passed chloroplast and mitochondrial filtering. All sequence data were uploaded to NCBI and accessible with project number PRJNA1095190 (https://www.ncbi.nlm.nih.gov/sra/PRJNA1095190).

**Fig. 4:**
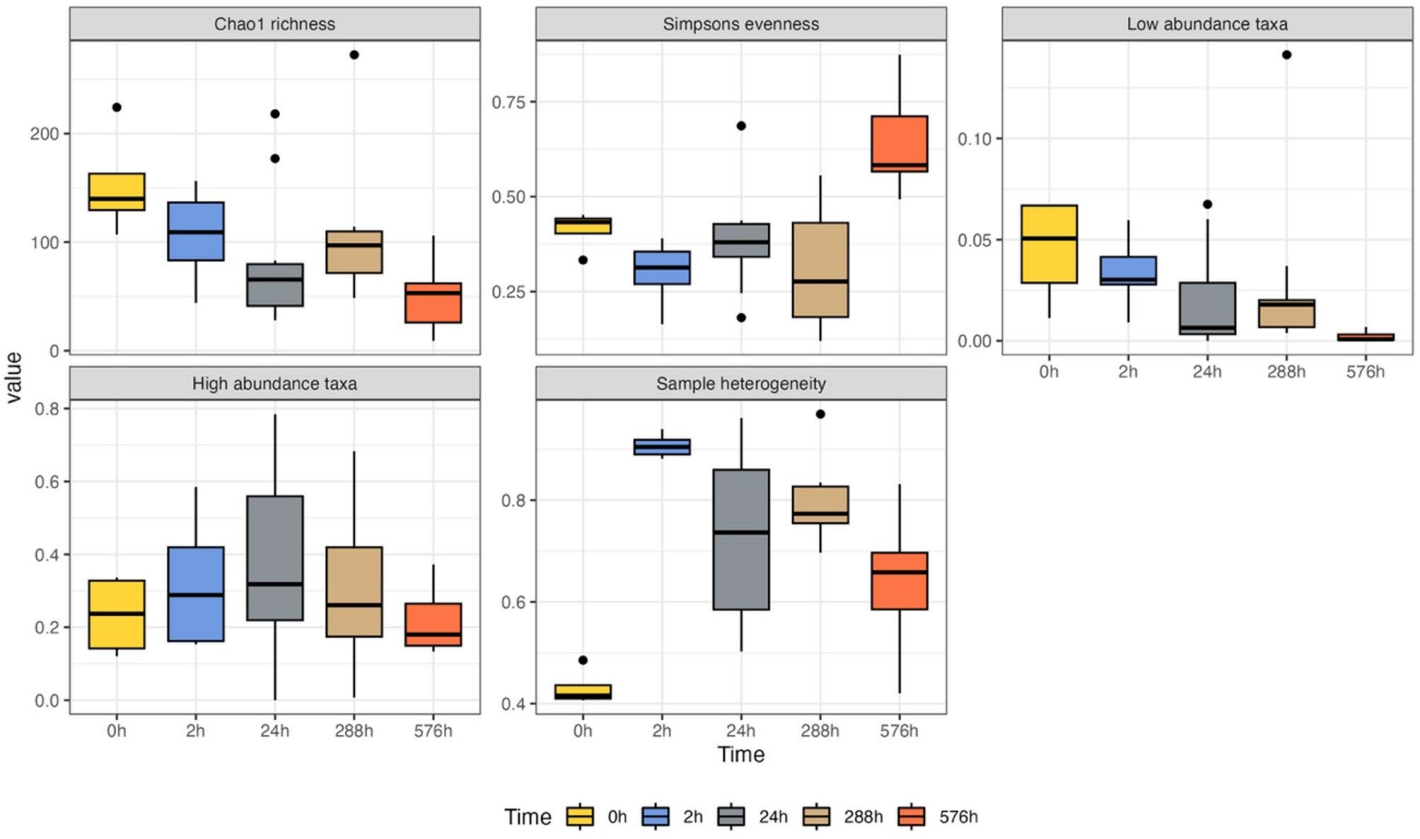
Comparison of diversity indices and sample heterogeneity among sampling times in Experiment 2. Legend annotation and Y-axis values refer to Fig. 1.

Chao1 species richness (Kruskal-Wallis rank sum test: *p* < 0.01, df = 4, χ^2^ = 14.1), Simpsons species evenness (Kruskal-Wallis rank sum test: *p* < 0.01, df = 4, χ^2^ = 22.4), relative proportion of low abundance taxa (Kruskal-Wallis rank sum test: *p* < 0.01, df = 4, χ^2^ = 20.1), and sample heterogeneity (Kruskal-Wallis rank sum test: *p* < 0.01, df = 4, χ^2^ = 25.7) changed significantly over time (Fig. 4). On the other hand, there were no significant differences in relative proportion of high abundance taxa amongst individuals over time (Kruskal-Wallis rank sum test: *p* = 0.50, df = 4, χ^2^ = 3.39) (Fig. 4). Significant differences from the beginning (0 h) were observed in Simpson’s evenness and sample heterogeneity as soon as in two hours (Table 3). Chao1 richness showed significant deviation after 24 h. showing significant differences between 0 h and all other time periods (Table 3).

**Table 3:** Experiment 2 diversity indices pairwise Wilcoxon rank sum test p-values.

		Chao1 richness	Simpson’s evenness	Low abundant taxa	High abundant taxa	Sample heterogeneity^1^
0 h	2 h	0.19	0.04*	0.30	0.45	< 0.01*
	24 h	0.05*	0.37	0.11	0.54	< 0.01*
	288 h (12d)	0.05*	0.30	0.19	0.64	< 0.01*
	576 h (24d)	< 0.01*	< 0.01*	< 0.01*	1.00	< 0.01*

^1^. Tested by PERMANOVA pairwise.

**Fig. 5:**
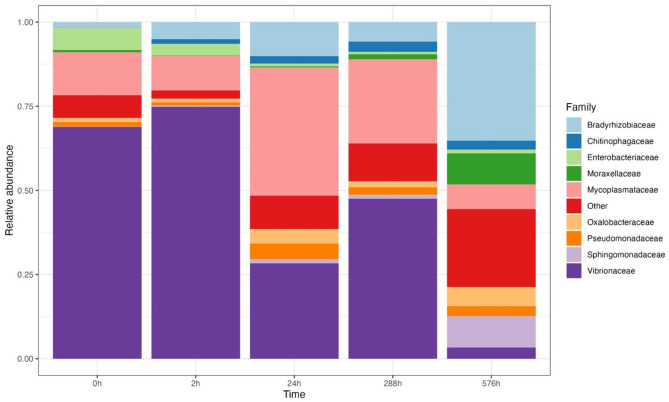
Relative abundance of the top ten most abundant families across time periods in experiment 2, coloured by family (Other: rare families with detection level < 0.01).

The most common microbiome families in experiment 2 were Vibrionaceae, Mycoplasmataceae, and Bradyrhizobiaceae (Fig. 5). The microbiome composition dissimilarity between 0 h and 576 h could be mostly explained by the changes in Vibrionaceae (SIMPER: 30%, *p* = 0.03) and Bradyrhizobiaceae (SIMPER: 17%, *p* = 0.01). Over time, the relative abundance of Vibrionaceae (GLM: *p* < 0.01, coefficient = −0.001, AIC = 30.506) decreased significantly, and Bradyrhizobiaceae (GLM: *p* < 0.01, coefficient = 0.0004, AIC = −50.765) increased significantly.

### Comparisons between experiment 1 and experiment 2

Experiment 1 and experiment 2 showed similar species diversity and richness but different species evenness at the beginning of two experiments (0 h) (Table 4). For species diversity, significant differences between two experiments showed at 2 h and 24 h but not at 288 h and 576 h. For species richness, the differences between two experiments showed at 2 h and persisted to the end. For species evenness, only the beginning (0 h) and the ending (576 h) time point had significant differences between two experiments.

**Table 4:** Kruskal-Wallis rank sum tests between experiment 1 and experiment 2, p-values.

	Chao1 richness	Simpson’s evenness
0 h	0.20	0.02*
2 h	< 0.01*	0.26
24 h	0.02*	0.19
288 h (12d)	0.02*	0.97
576 h (24d)	< 0.01*	< 0.01*

## Discussion

Our study provides valuable insights into post-mortem changes in the gut microbiome of large commercial fish and as such, inform the development of sampling protocols for microbiome analyses in fisheries more generally. Firstly, we identified significant changes in the gut microbiome of skipjack tuna as early as 24 h after death. Given that gut samples from large commercial fish are normally collected days or weeks after capture, our findings indicate that this delay can significantly affect the gut microbiome composition, leading to potentially unreliable conclusions. Secondly, we identified specific bacterial families, such as Bradyrhizobiaceae and Vibrionaceae, that may serve as indicators of gut microbiome degradation in skipjack tuna. More precisely, a high relative abundance of Bradyrhizobiaceae and a low relative abundance of Vibrionaceae suggests a state of gut microbiome degradation. In cases where the time between capture and sampling is unknown, the relative abundance of Bradyrhizobiaceae and Vibrionaceae could be used to indicate the level of degradation: a high abundance of Vibrionaceae and low abundance of Bradyrhizobiaceae indicates a fresh, undegraded sample, whereas a low abundance of Vibrionaceae and high abundance of Bradyrhizobiaceae suggests that degradation has occurred.

We observed a significant decrease in the diversity of the gut microbiome in skipjack tuna over time post-mortem, suggesting a degradation of the gut microbiome community. When analysing gut microbiome samples from different skipjack tuna individuals at various time points, we found that while each fish had a unique gut microbiome composition, the patterns of change over time were consistent across individuals in both experiments. Our findings suggests that the timing of sample collection and preservation significantly affects the reliability of gut microbiome data. Both experiments underline the importance of preserving gut samples within the first 24 h post-mortem. Significant differences in biodiversity, particularly in species richness, were observed beyond this timeframe. Therefore, we proposed that 24 h post-mortem is a critical threshold for obtaining reliable gut microbiome data in large commercial fish. Samples collected and preserved beyond this period are likely to yield unreliable results and should be interpreted with caution.

Our findings contrast with post-mortem studies in mammals, which have reported relatively stable gut microbiomes within comparable timeframes^15,18,31^. For instance, the gut bacterial communities of Atlantic bottlenose dolphins (*Tursiops truncatus*) showed only minor compositional changes, even when the hosts were moderately decomposed^31^, suggesting that the onset and rate of post-mortem microbiome degradation may be species-specific and context dependent. The faster shifts observed in our study may be attributed to several factors, including differences in host physiology (e.g., high metabolic rates in tunas)^32,33^, environmental conditions (e.g., temperature or salinity exposure post-capture)^34,35^, or differences in microbial community composition between taxa^36^. These findings highlight the importance of validating preservation windows for each species and environmental context, rather than relying on assumptions drawn from studies in other taxa.

Our analysis identified two families, Vibrionaceae and Bradyrhizobiaceae, that underwent the most significant changes over time. Vibrionaceae is prevalent in the gut microbiome of many marine fishes, including tuna^3,4,20,37,38^ while Bradyrhizobiaceae is rarely found in marine fish. The dominance change of these two families may be related to the competition between them, which has been found previously^39^. Meanwhile, changes in nitrogen concentration following capture from the ocean may also result in the increase of Bradyrhizobiaceae, because one of its important functionality is to fix atmospheric nitrogen^40^.

The significant changes in the two bacterial families over time may indicate their sensitivity to sampling and preservation time. Studies that report a high relative abundance of Bradyrhizobiaceae and/or low relative abundance of Vibrionaceae should consider the potential impact of sampling and preservation time. The reliability of such studies should be carefully evaluated. For example, one study found a higher relative abundance of Bradyrhizobiaceae in frozen fish gut samples compared to fresh samples^41^, supporting Bradyrhizobiaceae as an indicator of gut microbiome degradation. Moreover, although the relative abundance of other bacterial families did not change as significant as that of Bradyrhizobiaceae and Vibrionaceae, the patterns observed in these families could also assist in estimating the level of microbiome degradation, alongside Bradyrhizobiaceae and Vibrionaceae. For example, Gadoin et al. (2021) observed a low relative abundance of Vibrionaceae but a high relative abundance of Mycoplasmataceae in the gut of skipjack tuna, which is similar to the microbiome composition observed 24 h post-mortem in inter-individual experiment of our study. In Gadoin et al.‘s (2021) study, fish were frozen and fish guts were extracted after landing, similar to the scenario in inter-individual experiment of our study, suggesting that the gut microbiome degradation might have occurred in Gadoin et al.‘s (2021) study.

### Implications

The degradation of gut microbiome in wild free-living fish can significantly affect the reliability of gut microbiome analyses. However, the timing and patterns of such degradation remain unclear. Our study established a critical time point for preserving gut samples, 24 h post-capture, to recover the “true” gut microbiome composition. Therefore, we recommend that gut samples be preserved properly as soon as possible for microbiome analyses. Samples preserved beyond 24 h are likely to yield unreliable results and implications, potentially introducing confounding factors that obscure true correlations between the gut microbiome and external variables. For instance, the failure to detect a correlation between the gut microbiome of skipjack tuna in relation to ENSO events in a recent study^21^ is likely to have been influenced by post-mortem microbiome degradation given that gut samples were extracted after landing and a low relative abundance of Vibrionaceae was observed^21^. The importance of implementing reliable sampling and preservation strategies has also been observed in other studies on wild marine fish. For example, contamination and microbial changes occurring during onboard handling and pumping procedures caused shifts in bacterial diversity and composition in Atlantic mackerel stored in refrigerated seawater tanks^19^. Together, these examples reinforce the need for robust preservation protocols to minimize microbiome alteration during fish capture and processing. Without such strategies, gut microbiome profiles from wild marine fish may reflect handling conditions rather than the host’s original microbial community.

When the time frame between fish capture and gut sampling is unclear, a low relative abundance of Vibrionaceae and/or a high abundance of Bradyrhizobiaceae can indicate microbiome degradation. Along with the composition of other bacterial families, our study proposes a preliminary reference pattern for gut microbiome composition changes, which could assist in evaluating the reliability and level of degradation in the gut microbiome of marine pelagic fish. Our study has implications for other large marine fish because the changes in the gut microbiome are consistent across individuals and other larger fish with higher metabolic rate are likely to have more significant post-mortem changes.

Future research could explore predictive models utilizing key microbiota species and biodiversity indices to estimate gut microbiome degradation levels, thereby improving the accuracy of microbiome analysis and mitigating confounding factors. Additionally, such models could aid in utilizing samples preserved for longer periods to infer the “true” gut microbiome of living hosts.

## Conclusions

Our findings contribute to a better understanding of microbiome composition in wild-caught large marine fish and highlight the need for representative sample collection. While microbiome changes after death are generally assumed, we provide strong evidence that the timing and extent of such changes can be substantial, and even predictive, in wild-caught marine fish and warrants further investigation. This knowledge gap limits the development of robust sampling protocols, which are essential for ensuring repeatability and data reliability of gut microbiome studies. Our results demonstrate that preserving fish gut microbiome samples promptly and appropriately is crucial for achieving reliable results. The decrease in relative abundance of Vibrionaceae and increase in Bradyrhizobiaceae can serve as potential indicators for the gut microbiome decomposition. Based on our findings, preserving samples in nucleotide preservatives such as RNA*later* within a two-hour timeframe is recommended to obtain the most representative microbiome composition. Conversely, samples preserved after 24 h may not achieve reliable results.

## Method

### Experimental design

**Fig. 6:**
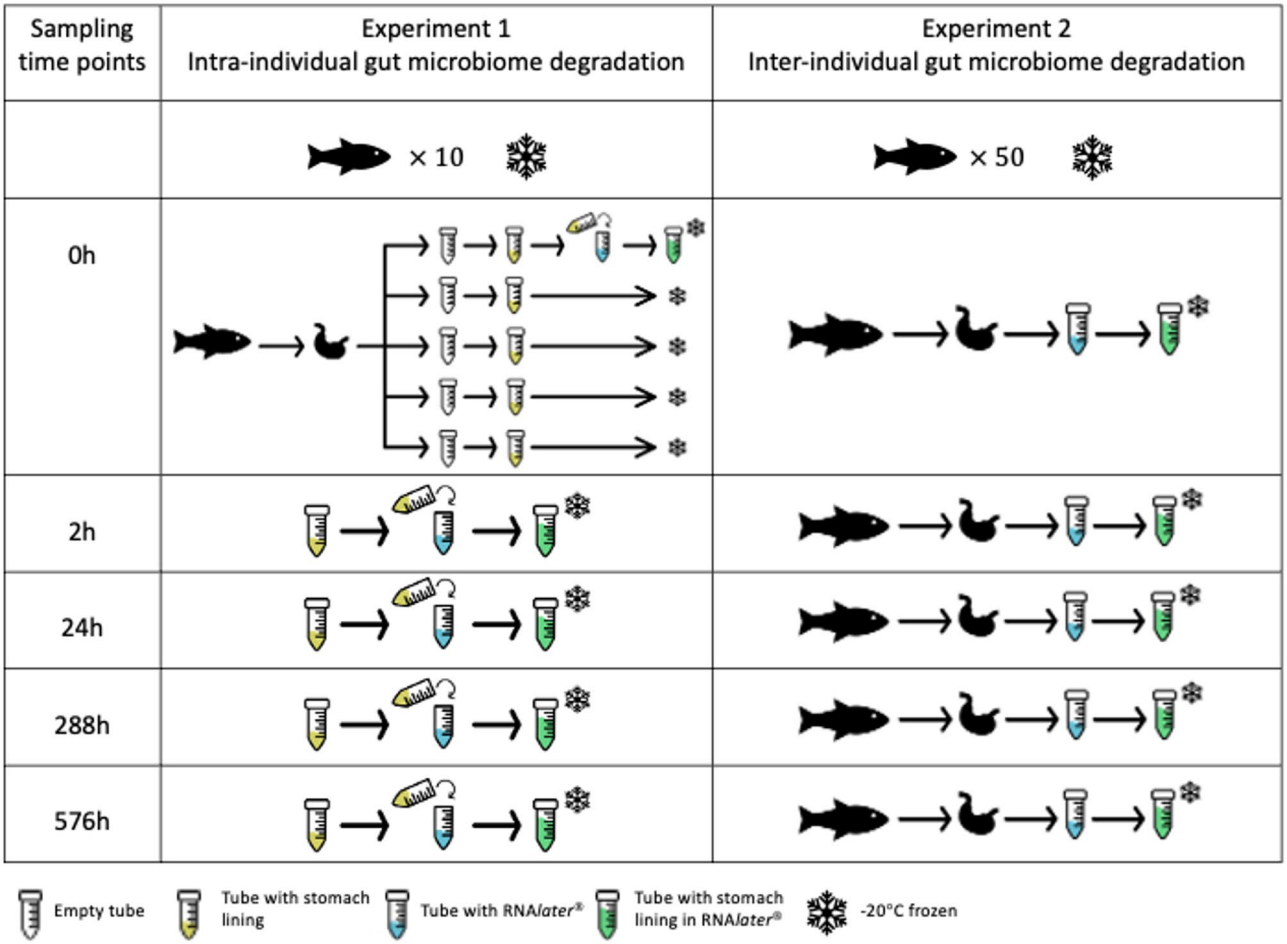
Illustration of experimental design. 0 h: immediately after the catch; 2 h, 24 h, 288 h, 576 h: 2 h, 24 h, 12days, and 24 days after the catch.

Two experiments were designed to investigate the change in the gut microbiome of skipjack tuna (*Katsuwonus pelamis*) at five distinct time points (immediately after the catch, 2 h, 24 h, 12 days, and 24 days after the catch, Fig. 6). In the first experiment, ten skipjack tuna of a similar size (TL ± S.D. = 618 ± 9.20 mm) were caught by pole and line methods on the same day in the Solomon Islands Exclusive Economic Zone (EEZ) (0911.540 S, 15752.780E) in September of 2022. After capture, these fish were temporarily stored in an iced brine bath to maintain freshness. The gut linings of each fish were extracted within 30 min of capture using sterilized scissors and forceps and homogenized in Milli-Q water using a sterilized single-use plastic spoon. Then, the homogenized gut sample of 5 mL of gut sample from each fish was aliquoted into five separate tubes each. This aliquoting approach was used to avoid repeated freeze–thaw cycles, which could confound the effects of preservation time on microbiome composition^16^. The contents of one tube of gut lining from each fish was immediately transferred into a separate 15 mL tube containing 10 mL of RNA*later* to preserve DNA and RNA, which represents samples from 0 h time point (immediately after the catch). The remaining four tubes of gut lining from each fish were kept frozen at −20 °C in a freezer. Then, one tube of gut lining from each fish was defrosted at room temperature and under shade for 30 min at either 2 h (2 h), 24 h (24 h), 12 days (288 h), and 24 days (576 h) after the catch and transferred into a separate 15 mL tube containing 10 mL of RNA*later* to preserve DNA and RNA. This resulted in a total of 50 gut samples for Experiment 1. Even though the manufacturer’s protocol suggests to keeping the samples in RNA*later* for 24 h at 4 °C, some studies found that this stabilization led to greater reductions in DNA and RNA yields^42,43^. We therefore chose to reduce the risk of this happening by placing samples at −20 °C immediately after being transferred into RNA*later*. One negative control was also included in each sampling event, which consisted on keeping a sterile 50 mL falcon tube with 10mL of RNA*later* uncapped on the work bench for each sampling event, capped, and then stored at −20 °C.

In the second experiment, 50 skipjack tuna with similar size (TL ± S.D. = 480 ± 19.89 mm) were caught by pole and line methods on the same day in the Solomon Islands EEZ (0847.000 S, 15714.160E) in September of 2022. Fish were kept frozen using a brine bath right after the catch. At each sampling time as per the first experiment 1 (0, 2 h, 24 h, 12 days, and 24 days), ten fish were randomly selected and defrosted at room temperature and under shade for up to two hours. Then, the gut linings of the ten fish were extracted individually using sterilized scissors and forceps. Approximately 20 mL of the stomach lining from each fish was homogenized using a sterilized single-use plastic spoon and transferred into a separate 50 mL falcon tube containing 30 mL of RNA*later* to preserve DNA and RNA. This resulted in a total of 50 gut samples for Experiment 2. Gut lining samples in RNA*later* were then frozen at−20 °C. One negative control tube with 10 mL of RNA*later* was included in each sampling event, kept open from the beginning of each sampling event, capped, and then stored at −20 °C.

All samples were placed in dry ice and shipped for metabarcoding analysis to the University of Canberra within two shipping days in October 2022 and kept frozen at −20 °C before further molecular analysis.

### DNA extraction and amplification

Frozen samples from both experiments were thawed at room temperature before DNA extraction. The gut lining samples were diluted by adding an equal volume of ice-cold PBS right before the DNA extraction according to RNA*later* manufacturer’s instructions, to enable efficient centrifugation process (Thermo Fisher Scientific, Australia). The diluted samples were centrifuged at 7,850 rpm for 10 min. After centrifugation, the supernatant was discarded, and the pellet was retained for DNA extraction.

DNA extraction was performed using the ZymoBIOMICS MagBead DNA/RNA Purification protocol with minor modifications. Initially, 180 µL ZymoBIOMICS^™^ DNA/RNA shield and 20 µL Proteinase K was added to the pellet, followed by overnight incubation at 56 °C for digestion. Subsequently, 1 mL DNA/RNA lysis buffer was added to each sample, and the manufacturer’s protocol was followed for the remaining steps of the extraction process. The final DNA was eluted in 50 µL ZymoBIOMICS^™^ DNase/RNase-Free Water. The extracted DNA was assessed for yield and quality using a Nanodrop 2000 Spectrophotometer and then stored at −20 °C.

### Library preparation and metabarcoding sequencing

Microbiome diversity was examined by amplifying the V3-V4 region of the 16S rRNA gene region using primers with Illumina overhangs Pro341F (5’ - CCTACGGGNBGCASCAGTCGTCGGCAGCGTCAGATGTGTATAAGAGACAG) and Pro805R (5’ - GACTACNVGGGTATCTAATCCGTCTCGTGGGCTCGGAGATGTGTATAAGAGACAG)^44^ and prepared for metabarcoding analysis. Each sample was amplified using real-time PCR, wherein each reaction contained 5 µL of extracted DNA, 2.5 µL of Gold buffer (10X), 2 µL of MgCl_2_ (25 mM), 0.65 µL of dNTPs (10 mM), 0.2 µL of AmpliTag Gold Polymerase, 0.5 µL of Sybr Green (1/2000), 12.15 µL of Ultra-purified DNase/RNase-Free Denoised water, and 1 µL of each primer pair (10 µM) for a total volume of 25 µL. Each sample was amplified using three technical replicates in a QuantStudio™ 7 Pro (Thermo Fisher Scientific, Australia) with initial incubation at 50 °C for 2 min, initial denaturation at 95 °C for 10 min, 32 cycles of 95 °C denaturation for 15 s, 60 °C annealing for 30 s, 72 °C extension for 30 s with ramp temperature rate at 1.6 °C/s. Followed by a melt curve analysis from 60 °C to 95 °C was then completed with a ramp temperature rate of 0.15 °C/sec. Negative controls and microbiome community standard were included in each plate. Amplification-positive products were cleaned with AMPure XP beads using a 1:1.2 volume ratio.

Cleaned amplicons were then prepared for metabarcoding. Illumina DNA/RNA UD indexes (Set A) were ligated to cleaned amplicons by PCR. First, positive amplicons per sample were pooled together and briefly homogenised. Then single reactions per pooled sample were prepared with 5 µL of cleaned PCR products, 20 µL of Illumina Enhanced PCR Mix, 10 µL of IDT for Illumina DNA/RNA UD Indexes (Set A), and 15 µL of Ultra-purified DNase/RNase-Free Denoised water for a total volume of 50 µL. Each reaction was run in a QuantStudio™ 7 Pro (ThermoFisher Scientific, Australia) with an initial incubation for 68 °C for 3 min and 98 °C for 3 min, followed by 12 cycles of 98 °C for 45 s, 62 °C for 30 s, 68 °C for 2 min, and a final extension of 68 °C for 1 min. Two negative controls were included. Tagged amplicons were cleaned with AMPure XP beads as recommended by the manufacturer’s protocol, and the concentrations were assessed using Qubit HS Assay.

All amplicons were normalized and pooled to construct the final HTS library. The concentration of the final library was assessed using Qubit HS Assay, and the quality and length of the final library was checked on a 1.5% agarose gel. The final library was mixed with 25% PhiX according to the suggestions from Illumina, and sequenced on the Illumina MiSeq platform using a V3 2 × 300 bp (600 cycles) sequencing kit.

### Bioinformatics analysis

The generated FASTQ file was automatically demultiplexed and the primers and adapters were removed by the Illumina Local Run Manager. The analysis was then carried out in QIIME2 (version 2021.4.0)^45^. The demultiplexed FASTQ files were quality-evaluated, denoised, and filtered using DADA2 (1.22.0)^46^. The forward reads were truncated at 275 bases without trim at the 5’ end, and reverse reads were truncated at 225 bases without trim at the 5’ end. The maximum number of expected errors for quality filtering was two. Then the filtered forward and reverse reads were merged using DADA2 (1.22.0)^46^. Multiple sequence alignment of Amplicon Sequence Variants (ASVs) was performed using MAFFT^47^. Chimeras were also removed using DADA2 (1.22.0)^46^. Taxonomic information was assigned to each ASV against a curated SILVA (138) reference database^48^ with only 16 S rRNA V3-V4 region sequences. ASVs without phylum information or belong to chloroplasts or mitochondrial were removed using phyloseq package (version 1.38.0)^49^ and microbiome package (version 1.23.1)^50^ in R (version 4.1.2)^51^.

### Statistical analysis

All statistical analyses were performed using R (version 4.1.2)^51^ within RStudio (version 2022.2.0.443)^52^. Data visualization utilized the ggplot2 package (version 3.4.0)^53^.

For diversity analyses, data remained non-normalized, and indices were calculated using the microbiome package (version 1.23.1)^50^. Species richness was assessed using the Chao1 index^54^, and species evenness was tested using Simpson’s index^55^. The relative abundance of all low abundance taxa (i.e., < 0.2% relative abundance, < 20% prevalence) and all high abundant taxa (i.e., ≥ 0.2% relative abundance, >50% prevalence) were calculated^56^.

Normality of diversity indices was examined using the Shapiro-Wilk test^57^. In Experiment 1, all diversity indices followed a normal distribution (Additional file 2, Table 1), therefore analysis of covariance (ANCOVA) test was used to compare indices among time points while controlling for individual variances^58^. Tukey’s honestly significant difference (HSD) test was applied for pair-wise comparisons between 0 h and other time groups^59^ where significant differences were observed.

In Experiment 2, Kruskal-Wallis rank sum test^60^ was applied due to non-normal distribution of some diversity indices (Additional file 2, Table 1). Pairwise comparisons using Wilcoxon rank sum test^61^ were applied to compare the diversity indices between each time group and the beginning point (0 h) in Experiment 2.

For sample heterogeneity analyses, total sum normalization (TSS)^62^ was applied to both experiments using the phyloseq package (version 1.38.0)^49^. Sample heterogeneity changes across time were calculated using the microbiome package (version 1.23.1)^50^. Permutational multivariate analysis of variance (PERMANOVA) was performed with pseudo-F and 999 permutations to assess individual-level and time-related variances^63^.

Principal component analysis (PCoA) using Bray Curtis distance was conducted to identify outliers and evaluate the effects of time and individual variances. Comparisons between Experiment 1 and Experiment 2 were made for Chao1 species richness and Simpson’s species evenness at each time point.

The relative abundance of the top ten abundant families and genera was calculated, using the microbiome package (version 1.23.1)^50^. For each time point, the average relative abundances were calculated for the top ten abundant families and genera using the dpylr package (version 1.1.2)^64^. All rare taxa (relative abundance < 1%) were aggregated into one group “Other”. The important families which showed significant contribution to the dissimilarities between each time period were identified using similarity percentage (SIMPER) analysis in the vegan package^65^. Generalized linear models (GLM) were applied to important taxa to investigate changing patterns over time^66^. ANCOVA was conducted on important taxa to assess consistency among individuals^58^.

## Supplementary Information

Below is the link to the electronic supplementary material.

Supplementary Material 1

Supplementary Material 2

## Data Availability

Sequence data that support the findings of this study have been deposited in the National Center for Biotechnology Information (NCBI) with project number PRJNA1095190 (https://www.ncbi.nlm.nih.gov/sra/PRJNA1095190). The datasets supporting the conclusions of this article are included within the article and its additional files.
