# Supplementary material for: Impact of degradation and time of sampling on gut Microbiome composition in wild-caught marine fish
Source: Sci Rep. 2025 Aug 7;15:28947. doi: 10.1038/s41598-025-14683-9 (PMC12331957; doi:10.1038/s41598-025-14683-9)
Supplement: Supplementary file 2 — Supplementary Material 2 [file 41598_2025_14683_MOESM2_ESM.docx]

# **Impact of degradation and time of sampling on gut microbiome composition in wild-caught marine fish**

# Yufei Zhou, Alejandro Trujillo-González, Simon Nicol, Marion Boutigny, Roger Huerlimann, Stephen D. Sarre, Dianne Gleeson

# Additional file 2 statistical analysis results

| **Additional Table1. Results for Shapiro-Wilk normality test for alpha diversity indices** | | | | | | | | |
| --- | --- | --- | --- | --- | --- | --- | --- | --- |
|  | Experiment 1 | | | | Experiment 2 | | | |
|  | Chao1 richess | Simpsons evenness | Low abundance taxa | High abundance taxa | Chao1 richess | Simpsons evenness | Low abundance taxa | High abundance taxa |
| W | 0.97 | 0.97 | 0.96 | 0.98 | 0.98 | 0.96 | 0.78 | 0.98 |
| *p* | 0.31 | 0.33 | 0.12 | 0.65 | 0.62 | 0.21 | < 0.05 ** | 0.05 * |

| **Additional Table2. Kruskal-Wallis rank sum test for alpha diversity indices differences among individuals for Experiment 1** | | | | |
| --- | --- | --- | --- | --- |
|  | Chao1 richness | Simpsons evenness | Low abundance taxa | High abunance taxa |
| Df | 9 | 9 | 9 | 9 |
| χ^2^ | 20.1 | 22.3 | 17.6 | 25.3 |
| *p* | 0.01 ** | < 0.01 ** | 0.04 * | < 0.01 ** |

| **Additional Table3. PERMANOVA test with 999 permutations for beta diversity differences among fish individuals for Experiment 1** | | | | |
| --- | --- | --- | --- | --- |
|  | Df | R^2^ | F | Pr (>F) |
| Fish individual | 9 | 0.2 | 1.05 | 0.36 |
| Residual | 37 | 1.79 |  |  |
